# Rice Disease Detection: TLI-YOLO Innovative Approach for Enhanced Detection and Mobile Compatibility

**DOI:** 10.3390/s25082494

**Published:** 2025-04-15

**Authors:** Zhuqi Li, Wangyu Wu, Bingcai Wei, Hao Li, Jingbo Zhan, Songtao Deng, Jian Wang

**Affiliations:** 1 School of Computer and Control Engineering, Northeast Forestry University, Harbin 150006, China; 3056615581@nefu.edu.cn (Z.L.); rttt@nefu.edu.cn (H.L.); jingbo.zhan@nefu.edu.cn (J.Z.); 2 School of Computer Science, University of Liverpool, Liverpool L69 3DR, UK; wangyu.wu@liverpool.ac.uk; 3 School of Computer Science, Wuhan University, Wuhan 430072, China; weibc97@whu.edu.cn; 4 College of Information and Communication Engineering, Hainan University, Haikou 570228, China; dst@hainanu.edu.cn

**Keywords:** transfer learning, small object detection, rice disease detection, real-time mobile detection

## Abstract

As a key global food reserve, rice disease detection technology plays an important role in promoting food production, protecting ecological balance and supporting sustainable agricultural development. However, existing rice disease identification techniques face many challenges, such as low training efficiency, insufficient model accuracy, incompatibility with mobile devices, and the need for a large number of training datasets. This study aims to develop a rice disease detection model that is highly accurate, resource efficient, and suitable for mobile deployment to address the limitations of existing technologies. We propose the Transfer Layer iRMB-YOLOv8 (TLI-YOLO) model, which modifies some components of the YOLOv8 network structure based on transfer learning. The innovation of this method is mainly reflected in four key components. First, transfer learning is used to import the pretrained model weights into the TLI-YOLO model, which significantly reduces the dataset requirements and accelerates model convergence. Secondly, it innovatively integrates a new small object detection layer into the feature fusion layer, which enhances the detection ability by combining shallow and deep feature maps so as to learn small object features more effectively. Third, this study is the first to introduce the iRMB attention mechanism, which effectively integrates Inverted Residual Blocks and Transformers, and introduces deep separable convolution to maintain the spatial integrity of features, thus improving the efficiency of computational resources on mobile platforms. Finally, this study adopted the WIoUv3 loss function and added a dynamic non-monotonic aggregation mechanism to the standard IoU calculation to more accurately evaluate and penalize the difference between the predicted and actual bounding boxes, thus improving the robustness and generalization ability of the model. The final test shows that the TLI-YOLO model achieved 93.1% precision, 88% recall, 95% mAP, and a 90.48% F1 score on the custom dataset, with only 12.60 GFLOPS of computation. Compared with YOLOv8n, the precision improved by 7.8%, the recall rate improved by 7.2%, and mAP@.5 improved by 7.6%. In addition, the model demonstrated real-time detection capability on an Android device and achieved efficiency of 30 FPS, which meets the needs of on-site diagnosis. This approach provides important support for rice disease monitoring.

## 1. Introduction

Over half of the world’s population depends primarily on rice as their food supply, making it one of the most important global dietary staples [1]. Its significant production volume underscores rice’s importance in ensuring global food security and agricultural development. Statistics indicate that farmers lose an average of 37% of rice crops yearly to pests and diseases, with bacterial invasions being among the leading causes of reduced yield and crop fatality [2]. The detrimental effects of these pathogens extend beyond economic losses, posing serious threats to the environment and ecosystems. Thus, timely and efficient identification and management of these diseases is critical for farmers [3].This study aimed to detect three common rice diseases: bacterial spot disease, blast disease, and brown spot disease. Bacterial spot disease manifests as a watery spot on the tip or edge of the leaf, which eventually turns yellow and dry. Rice blast forms gray-centered spots with reddish-brown edges on leaves, nodes, and ears, and in severe cases, it kills the plant. Brown spot is characterized by dark brown to reddish brown round spots on leaves surrounded by yellow halos, which can lead to early leaf defoliation and yield reduction in severe cases. These diseases have a serious impact on rice yield and quality, and timely detection is essential for effective control. While traditional manual detection methods [4] are effective, they are time-consuming, labor-intensive, and prone to misdiagnosis. By contrast, deep learning-based methods have demonstrated exceptional performance in agricultural pest detection, offering higher accuracy and applicability [5].

Research by Conrad et al. [6] and Chen et al. [7] underscored the effectiveness of CNNs in detecting general rice diseases, highlighting their broad applicability. However, they identified a consistent challenge: the dependency on extensive, well-annotated datasets. This reliance poses a significant bottleneck to the scalability and practical applicability of CNN-based disease detection methods, underscoring a crucial area for future innovation and exploration. Transfer learning, which leverages pretrained networks to circumvent the need for large datasets (Shrivastava et al. [8]; Ahmed, K. et al. [9]), is seen as a solution to this challenge. While this strategy has shown promise, its application breadth across various uncommon rice diseases remains an area for further investigation. Li et al. [10] explored specialized methods for specific diseases, uncovering the profound potential of targeted diagnostics. The work of Ahmed, K. et al. [9] extended this exploration by integrating CNNs with traditional machine learning to enhance the performance in complex disease manifestations. Research by Daniya et al. [11] and Haridasan et al. [12] showcased innovations in CNN architectures and training strategies, offering new avenues to improve accuracy. However, their focusing primarily on classification accuracy overlooked the critical aspect of symptom localization, which is equally vital for comprehensive disease management. Emerging research has begun addressing these limitations by integrating novel architectural improvements and attention mechanisms to enhance accuracy and efficiency. Li, D.S. et al. [13] used an RCNN to propose a video detection framework based on deep learning which could quickly detect results and was better than the SOTA model at that time. Ma, N. et al. [14] greatly improved the quantity detection ability in the case of severe seed adhesion by introducing contribution convolutional layers and a deformable attention mechanism. Latif et al. [15] and Rahman et al. [16] contributed to this narrative. Patil et al. [17], Zhang et al. [18], and Jain et al. [19] introduced methods to optimize a CNN’s efficiency and mobile deployment, meeting the critical need for real-time, on-site diagnostics. However, coalescing these innovations into a cohesive framework to balance performance and computational efficiency remains an open area of research.

**Figure 1:**
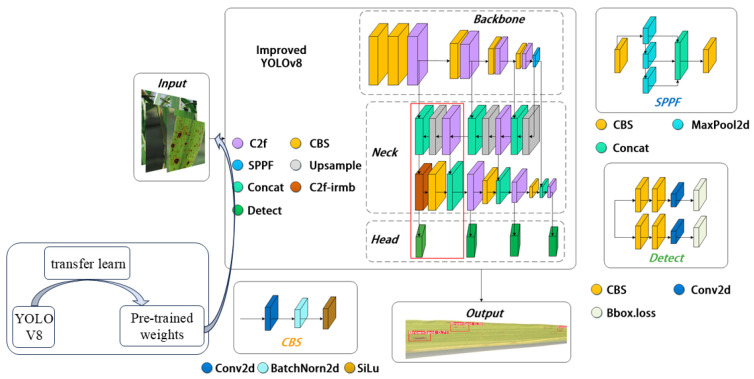
Improved YOLOv8 model structure diagram.

Moreover, research on other crops has further expanded the application of deep learning techniques. For example, Firnando, F.M. et al. [20] and Aldhyani et al. [21] highlighted the effectiveness of CNN-based architectures in detecting diseases in a variety of crops. Similarly, Rajamohanan et al. [22] demonstrated the adaptability of YOLO models to specific field datasets, optimizing performance for crop-specific diseases. While these studies provide valuable insights into the application of deep learning in plant disease detection, many focused on classification tasks or required significant computational resources. Enunice, J. et al. [23] discussed the need for efficient models in agricultural applications, especially those deployed in the field. Our approach, using the TLI-YOLO model, addresses the limitations of computational efficiency while maintaining high accuracy in real-time disease detection on mobile platforms. The research framework diagram for this study is shown in Figure 1.

In this paper, focusing on the challenges previously discussed, a novel object detection method based on transfer learning and an enhanced YOLOv8 algorithm are designed to address these difficulties. This study makes contributions in the following areas:

- The use of transfer learning to pretrain model parameters significantly reduces the need for extensive dataset collection and training time;
- An additional small object detection layer is added to the feature fusion layer, incorporating the iRMB attention mechanism into the C2f module. Combining feature maps of varying depths and integrating depthwise separable convolutions to preserve spatial integrity effectively enhances the detection capabilities for small object features and the efficiency of computational resource utilization;
- The WIoU is used in place of the CIoU as the loss function to improve the model’s generalization capability and balance the regression impact of samples of varying quality.
- By deploying the model on embedded devices, real-time detection of leaf diseases is achieved, aiding in the formulation of agricultural decisions and disease prevention strategies.

## 2. Related Work

### 2.1. Rice Disease Detection

Object detection algorithms based on deep learning, employing convolutional neural networks for feature extraction, are distinguished by their rapid processing, precise outcomes, and robust ability to generalize. These methodologies generally fall into one of two classifications, namely two-stage detection algorithms, such as Faster R-CNN [24], and one-stage detection algorithms, like YOLO [25]. While one-stage detection methods may not reach the precision levels of two-stage detection strategies, they offer higher detection speeds and the ability to perform real-time detection. Object detection methods can generally be classified into two categories: one-stage and two-stage detectors. In two-stage methods like Faster R-CNN, the first stage generates region proposals, and the second stage refines these proposals and classifies them, often yielding higher accuracy at the cost of slower speeds. By contrast, one-stage methods like YOLO bypass the region proposal stage, performing detection and classification in a single step. This approach is faster and more suitable for real-time applications, though it may sacrifice some accuracy compared with two-stage methods. Given our focus on real-time rice disease detection, we opted for a one-stage method to meet the requirements of real-time processing. Therefore, this study opts for one-stage object detection algorithms for real-time disease detection.

The You Only Look Once (YOLO) family has undergone several iterations since its initial release in 2016, each of which has brought significant architectural and performance improvements. As a pioneering work, YOLOv1 transformed object detection into a single-stage process for the first time, greatly improving the detection speed. YOLOv2 introduced batch normalization and anchor boxes, while YOLOv3 adopted multi-scale prediction and a residual network structure. YOLOv4 achieved a better balance between accuracy and speed by introducing CSPDarknet53 as the backbone network and PANet as the neck structure, while integrating a variety of data enhancement techniques. YOLOv5 simplified the model structure and provided different scale versions (s/m/l/x), making it easier to deploy and apply. YOLOv6 and YOLOv7 strengthened inference efficiency and architecture optimization, respectively. In YOLOv8, a more modern design concept was adopted, including an anchor-independent detection head and a C2f module to support multiple tasks, such as instance segmentation and pose estimation. With ongoing improvements in computing performance, deep learning-based disease detection algorithms have made significant progress, enhancing both detection performance and speed [26]. Jiang et al. [27] developed a detection technique for four types of diseases using a method based on 10-fold cross-validation with SVMs and CNNs, achieving an average accuracy of 96.8% for disease detection. However, this model struggles in complex detection environments. Additionally, Anami et al. [28] proposed a real-time rice disease detection algorithm based on a pretrained VGG-16 CNN model capable of detecting up to 12 different categories, with an average accuracy of 92.89%. Yet, this method shows low generalization and performs unstably under adverse weather conditions, thus revealing certain limitations.

Recent advancements in plant disease detection have made significant use of deep learning techniques to improve classification accuracy and real-time detection capabilities. For instance, the study by Sajitha, P. et al. [29] achieved a high classification accuracy of 96% on real-time images, demonstrating the power of YOLO architectures for multi-class plant disease detection. Similarly, Ritharson, P.I. et al. [30] applied deep learning models for the classification of rice leaf disease subtypes, achieving an impressive 99.9% accuracy using transfer learning and deep features.

Further studies, such that by Kaur, A. et al. [31], have employed transfer learning using pretrained CNN models like VGG16 and InceptionV3 to classify rice leaf diseases with high accuracy. Additionally, Dubey, R.K. et al. [32] and Stephen, A. et al. [33] have shown that incorporating advanced feature selection and deep learning architectures can improve both accuracy and robustness in rice leaf disease detection tasks.

### 2.2. YOLO

**Figure 2:**
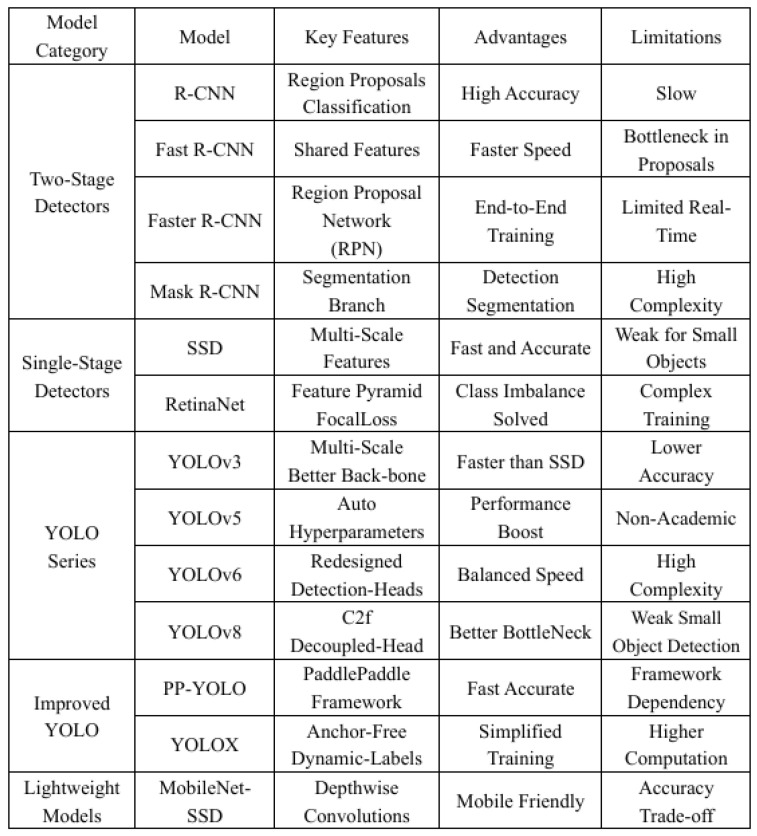
Comparison and analysis of major object detection models.

Wu et al. [34] made modifications to the YOLOv5 network architecture and introduced the YOLOV5-GHOST model for detecting vehicles and their distances in the CARLA environment. This modification effectively lowered the computational burden. Wang et al. [35] refined the YOLOv5 algorithm by adjusting the hyperparameters for transfer learning, experimenting with different network structures, and implementing detection head search techniques, leading to improved detection performance and reduced algorithm complexity. Zhu et al. [36] proposed the TPH-YOLOv5 model, which incorporates a Transformer-based predictive head into the YOLOv5 framework. This integration, combined with a convolutional block attention mechanism and data augmentation strategies, enhanced detection accuracy in high-density scenarios. Zheng et al. [37] utilized GhostNet to optimize the computational efficiency and training speed of YOLOv5. By integrating the precise image segmentation capabilities of U2-Net, they applied this approach to crack detection in complex environments, achieving significant improvements in classification efficiency and reducing the false detection rate. Liu et al. [38] introduced a ship detection method based on YOLOv5 and GhostNet. The addition of the GhostNet module further optimized the feature extraction process, significantly boosting detection accuracy while reducing the generalized intersection ratio error. Figure 2 shows the classification, characteristics, advantages, and defects of representative models in object detection.

## 3. Methods

**Figure 3:**
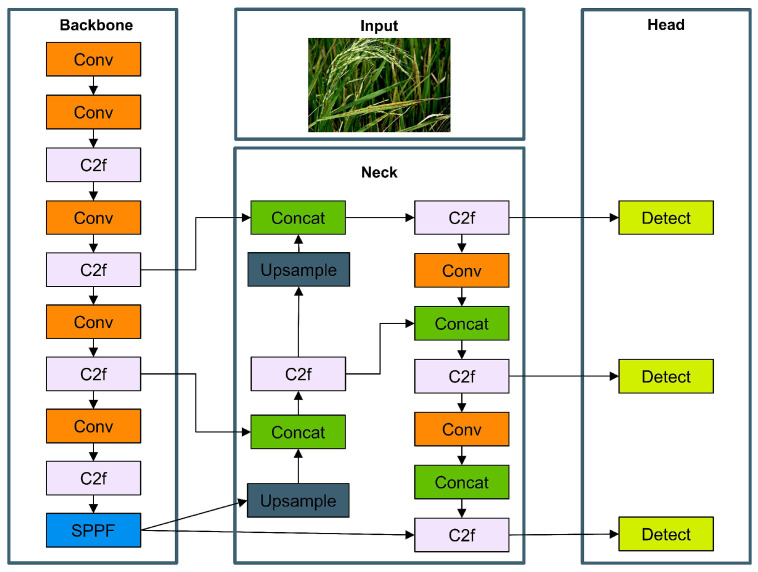
Standard YOLOv8 model structure diagram.

In this study, YOLOv8n was selected as the fundamental structure for several compelling reasons. As the latest iteration in the YOLO series, YOLOv8 introduces significant architectural improvements over its predecessors. The model employs a more efficient C2f module, which enhances feature representation by fusing deep and shallow features. This is particularly advantageous for rice disease detection, which requires simultaneous attention to texture details and the overall morphology. YOLOv8n, with only 3.2 M parameters, achieves a breakthrough in lightweight network design while maintaining detection accuracy, making it ideal for mobile deployment scenarios. Figure 3 displays the structure of the standard YOLOv8 model, consisting of three primary elements: the backbone, the neck, and the head. The backbone network utilizes convolutional layers with residual connections and bottleneck structures to extract image features while optimizing performance [39], with the C2f module serving as its basic building block. The neck network employs multi-scale feature fusion through the SPPF module, effectively integrating feature maps from different levels of the backbone. The head handles the ultimate detection objective with three size-varying detection heads optimized for targets of different scales. Unlike previous YOLO versions, which used traditional fully connected layer detection heads, YOLOv8 adopts a decoupled head design that separately predicts the object class and position, improving precision. Additionally, YOLOv8 integrates modern deep learning techniques such as adaptive feature aggregation and improved spatial pyramid pooling, making it more robust for small object detection and complex backgrounds. The comprehensive training and deployment ecosystem, supporting ONNX and NCNN conversion, further facilitates model deployment to mobile platforms.

### 3.1. Improved YOLOv8 Model

This article introduces a TLI-YOLO structure (Figure 1) designed to enhance the detection of rice diseases in natural environments, focusing on increasing both the speed and precision of identification. Initially, transfer learning is employed to allow the model to prelearn rich features of similar entities, significantly reducing training time and dataset requirements. Subsequently, a small-object detection layer (outputting high-resolution feature maps of 160 × 160), specifically aimed at detecting small objects 4 × 4 in size, is added. Furthermore, the iRMB attention mechanism is incorporated, merging it with the C2f module in the previously added small-object detection layer to form a new C2f_iRMB module. This module effectively extracts important feature information and enhances detail capture through the attention mechanism. This combination provides richer and more refined feature representations for rice disease detection, aiding in improving detection accuracy and efficiency. Finally, the original CIoU loss function is replaced with the WIoU v3 loss function, which evaluates the quality of prediction boxes more rationally through weighting. This allows for more accurate model optimization, especially in boundary box regression, effectively reducing detection errors for small objects and enhancing the model’s generalization ability.

### 3.2. Transfer Learning

**Figure 4:**
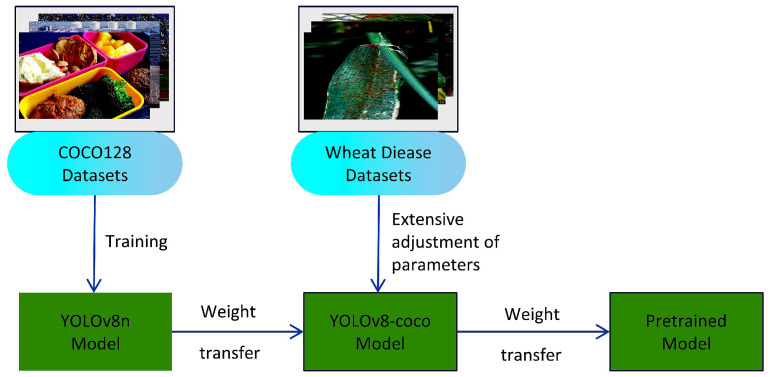
Transfer learning process diagram.

Deep learning is an algorithm driven by vast amounts of data, making training a model from scratch both time-consuming and costly. Transfer learning, a renowned method in machine learning, primarily involves pretraining a model on one or more tasks to learn certain features, which are then leveraged as a starting point for another task [40]. Specifically, this approach involves transferring the model weight parameters, which have been trained in a source domain, to a model designated for learning a new target. This not only allows developers to save time when training their models but also enhances the new model’s convergence speed [41], often resulting in a model with higher accuracy. Figure 4 illustrates the transfer learning process used in this study.

There are several reasons why we chose transfer learning for our rice disease detection model. First, the availability of large-scale pretrained models, such as those trained on the COCO128 dataset [42], provides a rich set of general features that are often applicable to a wide range of visual recognition tasks. By leveraging these pretrained weights, our model benefits from the ability to detect general features, such as edges, textures, and shapes, in the initial layers, features that are critical for distinguishing between healthy and diseased rice plants.

In our study, we initially pretrained the model on the COCO128 dataset as the source domain and further refined its ability to detect plant diseases, using a wheat disease dataset as the auxiliary domain. After two rounds of training, we transferred the learned weight parameters to the Transfer Learning Improved YOLO (TLI-YOLO) model, which was specifically designed for rice disease detection. This multi-domain pretraining approach ensures that our model benefits from a broad understanding of plant diseases before being fine-tuned for the specific task of detecting rice diseases.

### 3.3. Small Detection Layer

**Figure 5:**
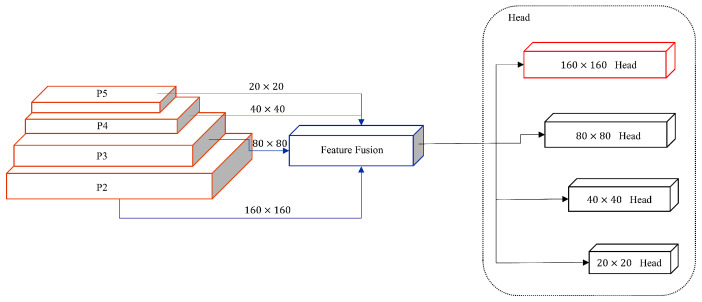
Macrostructure diagram after adding the small-object detection layer.

One key challenge in detecting small objects using YOLOv8 is the inherent difficulty deep feature maps face in learning the characteristics of small-sized samples, owing to the algorithm’s substantial downsampling factor. Addressing this, the research introduced a novel layer specifically tailored for small object detection. This layer enhances detection capabilities by merging both shallower and deeper feature maps, thereby facilitating more effective learning of small object features [43]. The red dashed box in Figure 2 illustrates the added components, including a C2f module, an upsampling layer, two Concat modules, an improved C2f_iRMB module, and a CBS module. Figure 5 showcases the macrostructure after adding a small target detection layer. The red rectangular box represents the added small object detection layer. Incorporating this layer allows the network to focus more on detecting small targets [44], thereby enhancing the accuracy of detecting small-scale rice diseases in images [45]. Although this increases computational demands and may slow down inference detection speeds, experimental tests showed that the GFLOPS only increased by 3.5, which is within the acceptable range. In the context of rice disease detection, precision and mean accuracy are our most crucial objectives.

### 3.4. C2f_iRMB Module

The core innovation of the Inverted Residual Mobile Block (iRMB) lies in its combination of the lightweight architecture of convolutional neural networks (CNNs) with the adaptive modeling capabilities of Transformers, creating an efficient neural network structure optimized for mobile platforms. Structurally, the iRMB is an improved design based on the inverted residual block originally introduced in MobileNetV2. It first expands low-dimensional features to a higher-dimensional space through 1 × 1 convolution, followed by depthwise separable convolution for spatial feature extraction, and it finally compresses the features back to low-dimensional representations via another 1 × 1 convolution. What distinguishes the iRMB from standard inverted residual blocks is its multi-branch architecture and attention mechanisms. The module includes (1) a primary branch using depthwise separable convolutions for spatial feature extraction; (2) a shortcut connection enabling cross-layer information flow and facilitating gradient propagation; and (3) a channel attention mechanism that recalibrates the importance of feature channels. Additionally, the iRMB incorporates dynamic convolution weight generation, allowing the module to adaptively adjust the convolution parameters based on the input content.

By integrating Transformer-like capabilities, the iRMB can capture long-range dependencies within images, offering a more comprehensive understanding of the spatial context. This is vital for rice disease detection, as the spatial distribution of symptoms can indicate both the type and severity of the disease. The unique combination of CNN and Transformer architectures in iRMBs provides a robust and efficient solution to rice disease detection on mobile devices.

**Figure 6:**
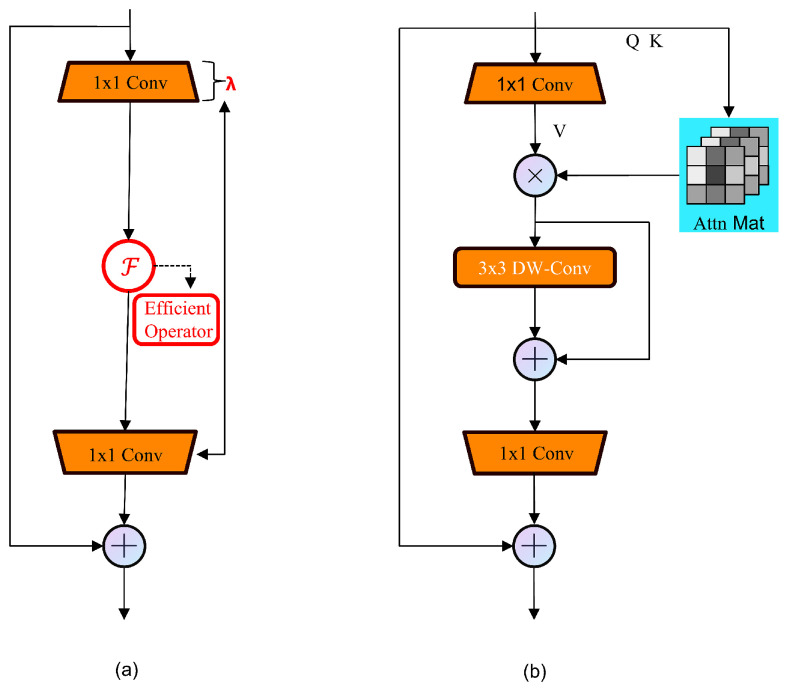
(**a**) Structure paradigm of MMB. (**b**) Structure paradigm of iRMB.

It offers high efficiency in environments with limited computational resources, making it suitable for dense prediction tasks on mobile devices [46]. The structural paradigm of iRMBs is illustrated in Figure 6b, while the iRMB is an extension of the Meta-Mobile Block (MMB), whose structural paradigm is shown in Figure 6a.

The MMB utilizes parameters such as the expansion ratio λ and the efficient function F to exemplify different modules. Starting with an image input $X\in R^{C\times H\times W}$ as an illustration, the MMB first uses an expansion ${\mathrm{MLP}}_{e}$ with the output/input ratio equaling λ to expand the channel dimension:

(1) $$X_{e}={\mathrm{MLP}}_{e}\left(X\right)\in R^{\lambda C\times H\times W},$$

Subsequently, the intermediate operator F enhances the features of the image. F is introduced as an efficient operator and is defined as follows:

(2) $$X_{f}=\left(X_{e}\right)F\in R^{\lambda C\times H\times W},$$

Ultimately, a shrinkage ${\mathrm{MLP}}_{s}$ with an inverted input/output ratio equaling λ is used to shrink the channel dimension:

(3) $$X_{s}={\mathrm{MLP}}_{s}\left(X_{f}\right)\in R^{C\times H\times W}.$$

where a residual connection is used to obtain the final output $Y=X+X_{s}\in R^{C\times H\times W}.$ This study develops a modern, efficient iRMB model built upon the MMB, incorporating efficient Window-MHSA (WMHSA) and Depthwise Convolution (DW-Conv) alongside a skip connection. This method effectively balances model cost and accuracy. The parameters and computations for Q and K in the WMHSA are based on the channel number squared. For efficient attention matrix computation, the non-expanded version of X is used, where Q = K = X. The enhancement, known as Expanded Window MHSA (EW-MHSA), is tailored for wider use and is established as follows:

(4) $$F\left(\cdot \right)=\left(\mathrm{DW}-\mathrm{Conv},\mathrm{Skip}\right)\left(\mathrm{EW}-\mathrm{MHSA}(\cdot )\right).$$

**Figure 7:**
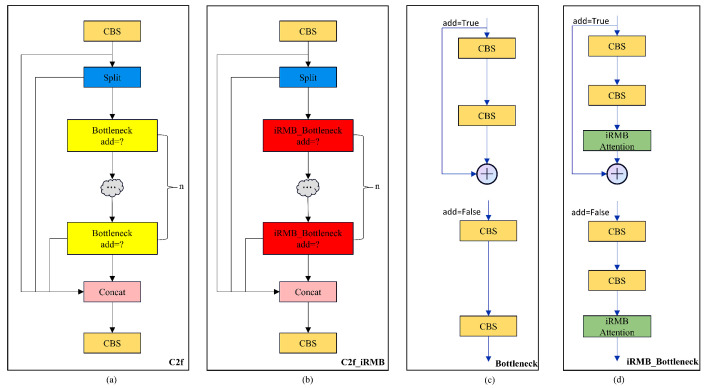
Structural diagrams of the C2f and C2f_iRMB modules. (**a**,**c**) for the original C2f module and (**b**,**d**) for the C2f_iRMB module.

The iRMB combines Depthwise Separable Convolution (DW-Conv) for capturing spatial features and a self-attention mechanism to grasp global dependencies among features. Its characteristics include the following. It employs Inverted Residual Blocks (IRBs), enhancing the processing of information flow by extending the traditional CNN’s IRB to attention-based models. This adaptation allows the model to handle long-range information more effectively. Such a design enables efficient operation on resource-constrained devices while maintaining or enhancing prediction accuracy:

1. By incorporating attention mechanisms, the iRMB can consider the entire input space when extracting features, thereby enhancing the model’s ability to comprehend complex data patterns, especially in processing visual and sequential data.
2. It abstracts a unified Meta-Mobile Block (MMB) from multi-head attention and feed-forward networks, combined with different expansion ratios λ and efficient operators F for design implementation. Integrating various operations into a unified framework in a plug-and-play manner enhances the model’s efficiency and flexibility, better balancing model complexity and computational efficiency [47].
3. After incorporating the iRMB attention mechanism, this paper integrates it with the traditional C2f module of YOLOv8, placing it subsequent to the CBS within the bottleneck. This arrangement allows the iRMB to further process the data with deep convolution as the CBS module is about to output, thereby optimizing computational resources and enhancing model efficiency. Figure 7 displays the structural diagrams.

### 3.5. WIoU Loss Function

Object detection performance critically depends on the loss function design, with bounding box losses playing a crucial role. While contemporary research focuses on enhancing bounding box fitting capabilities, excessive emphasis might not always improve detection outcomes, especially with low-quality samples in real-world datasets [48]. The weighted intersection over union (WIoU) addresses this by enhancing the traditional IoU metric through a weighting mechanism that emphasizes central object regions containing more discriminative features.

The WIoU exists in three distinct versions optimized for different aspects of bounding box regression. WIoU-v1 employs a Gaussian distribution weighting strategy, prioritizing pixels closer to the object’s center. WIoU-v2 introduces distance-based normalization, dynamically adjusting weights based on pixel positions, which is beneficial for irregular objects. WIoUv3, which was adopted in our study, introduces a dynamic, non-monotonic focusing mechanism that allocates more resources to challenging samples with occlusions or small objects. It combines positional weighting with a confidence-aware approach, integrating prediction confidence into the weighting function [49].

Compared with alternatives like the Smooth L1, CIoU, and GIoU, WIoUv3 offers superior performance for rice disease detection by providing balanced learning signals for different classes and effectively handling complex backgrounds. This paper innovatively replaces the CIoU loss function with WIoUv3, achieving more precise disease region localization while maintaining computational efficiency.

**Figure 8:**
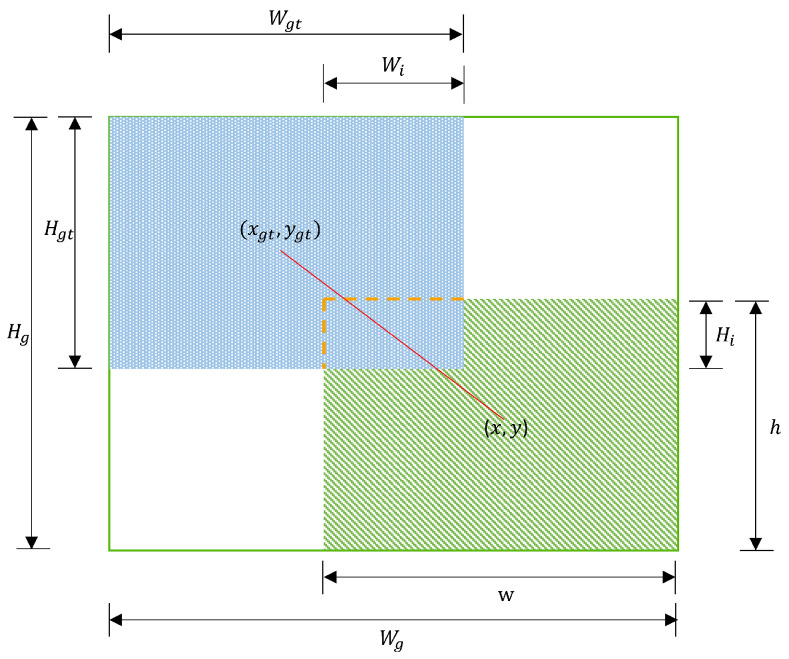
Boundary box regression model diagram.

In prior studies, the IoU stood out as a pivotal metric for gauging the degree of overlap between predicted anchor boxes and actual target boxes in object detection efforts. Figure 8 shows the bounding box regression model diagram. By converting the extent of this overlap into a proportion, it effectively counters any potential bias introduced by the size of the bounding boxes during the assessment phase. This approach ensures a fair learning process for models across both large and small objects when integrated into the Bounding Box Regression (BBR) loss function, fostering a more uniform training effect across objects of diverse sizes.

(5) $$\mathrm{IoU}=1-\frac{W_{i}H_{i}}{wh+W_{gt}H_{gt}-W_{i}H_{i}},L_{\mathrm{IoU}}=1-\mathrm{IoU}.$$

The generalization ability of the model is weakened when low-quality examples are present in the training set because geometric measurements like the aspect ratio and distance tend to magnify the penalties associated with these samples. Ideally, a loss function should mitigate the influence of geometric penalties, especially when there’s a significant overlap between anchor boxes and target boxes, ensuring minimal disruption during training to bolster the model’s generalization skills [50]. Building on this, we constructed a distance attention mechanism based on distance measures, resulting in WIoUv1, which incorporates a dual-layer attention mechanism:

(6) $$L_{\mathrm{WIoUv}1}=R_{\mathrm{WIoU}}L_{\mathrm{IoU}},$$

(7) $$R_{\mathrm{WIoU}}=\exp\left(\frac{{\left(x-x_{gt}\right)}^{2}+{\left(y-y_{gt}\right)}^{2}}{{\left(W_{s}^{2}+H_{s}^{2}\right)}^{*}}\right).$$

In this context, $R_{\mathrm{WIoU}}\in \left[1,e\right)$ significantly amplifies $L_{\mathrm{IoU}}$ for anchor boxes of standard quality. $L_{\mathrm{IoU}}\in \left[1,e\right)$ substantially reduces $R_{\mathrm{WIoU}}$ for high-quality anchor boxes and notably decreases its focus on the distance to the centroid in cases where the anchor box and target box overlap well. To prevent gradients that hinder convergence $W_{g}$, $H_{g}$ is detached from the computation graph (the superscript * signifies this operation).

Inspired by the focal loss [51], a monotonic focusing coefficient $L_{\mathrm{IoU}}^{\gamma *}$ was constructed:

(8) $$L_{\mathrm{WIoUv}2}=L_{\mathrm{IoU}}^{\gamma *}L_{\mathrm{WIoUv}1}\;\gamma >0$$

It can be observed that gradient enhancement correlates with $r=L_{\mathrm{IoU}}^{\gamma *}\in \left[0,1\right]$. Throughout the training, as $L_{\mathrm{IoU}}$ diminishes, so does the gradient boost, leading to a deceleration in the convergence rate during the advanced training phases. Hence, to mitigate this, the average of $L_{\mathrm{IoU}}$ is employed as a normalization factor:

(9) $$L_{\mathrm{WIoUv}2}={\left(\frac{L_{\mathrm{IoU}}^{*}}{L_{\mathrm{IoU}}}\right)}^{\gamma }L_{\mathrm{WIoUv}1}.$$

where $\bar{L_{\mathrm{IoU}}}$ denotes the moving average with a momentum of m. The dynamic updating of the normalization factor keeps the gradient gain $r={\left(\frac{L_{\mathrm{IoU}}^{*}}{L_{\mathrm{IoU}}}\right)}^{\gamma }$ at a relatively high level overall [52], addressing the problem of slow convergence as training progresses.

The anchor box’s outlier degree β is characterized by the ratio of $L_{\mathrm{IoU}}$ to $\bar{L_{\mathrm{IoU}}}$:

(10) $$\beta =\frac{L_{\mathrm{IoU}}^{*}}{L_{\mathrm{IoU}}}\in \left[0,+\infty \right)$$

For lower outlier values indicating high-quality anchor boxes, we allocate a diminished gradient boost, concentrating regression efforts predominantly on anchor boxes of standard quality. Larger negative gradients are prevented from being produced by low-quality instances by giving anchor boxes with a higher degree of outlier a lesser gradient increase. We constructed a non-monotonic focus coefficient using this principle and applied it to WIoUv1:

(11) $$L_{\mathrm{WIoUv}3}=rL_{\mathrm{WIoUv}1},\;r=\frac{\beta }{\delta \alpha^{\beta -\delta }}.$$

Here, r signifies the dynamic, non-monotonic focusing coefficient. This adaptability allows WIoUv3 to tailor its gradient gain allocation strategy to suit the current scenario effectively. The dynamic, non-monotonic focusing approach lessens the competitive edge of anchor boxes of higher quality while also diminishing the negative gradients from lower-quality examples, consequently enhancing the model’s effectiveness [53]. By eliminating the aspect ratio penalty found in the CIoU, the WIoU balances the effects of high- and low-quality anchor boxes on model regression, enhancing the model’s generalization ability and overall performance.

### 3.6. Deploying Mobile Platforms

This study developed a dedicated application designed for the identification of rice diseases, facilitating mobile detection on lightweight devices and enabling users to conduct real-time detection through the app. Our system offers two deployment options: an Android application performing on-device inference and a WeChat mini-program utilizing cloud-based inference. It is crucial to note that the mini-program does not process images locally; instead, it sends user-submitted images to our cloud servers, where the TLI-YOLO model is deployed, and then returns results to the user’s device. This cloud-based approach maintains a lightweight client while ensuring accurate detection capabilities across various devices, though it requires internet connectivity. This design addresses WeChat’s limitations regarding on-device deep learning deployment while maximizing accessibility.

**Figure 9:**
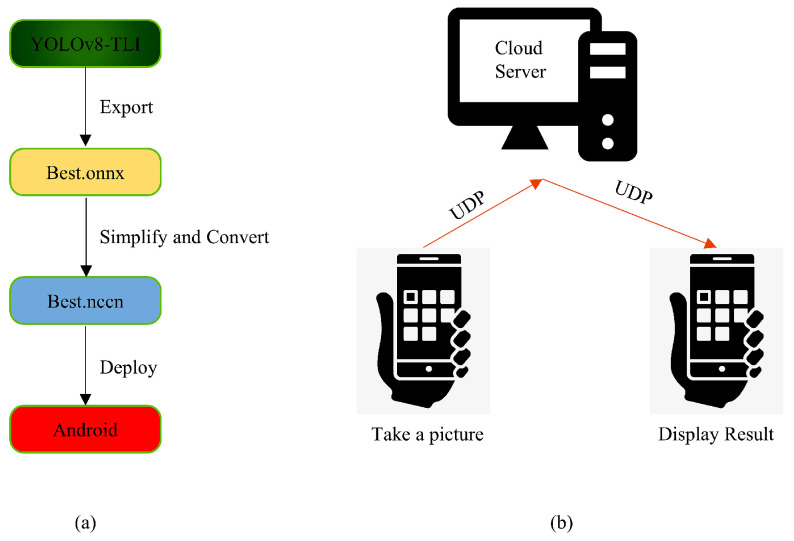
Mobile platform implementation diagram. (**a**) represents the model transformation process, and (**b**) shows the applet data transfer process.

The implementation process is illustrated in Figure 9a. The key steps include first converting the trained model to ONNX format for storage. ONNX provides a set of environment- and platform-independent standard formats, allowing models to interact across different frameworks and environments [54]. Secondly, the ONNX format model is converted into a file usable by NCNNs. An NCNN is a high-performance neural network forward-computing system designed for mobile devices that is quicker than any open-source framework now available on mobile CPUs and independent of external dependencies [55]. During the conversion from ONNX to an NCNN, due to the large size of the model, which could prevent conversion, this study simplified and merged the ONNX model before converting it to an NCNN. Lastly, the converted model is deployed on the Android platform for real-time detection, achieving satisfactory identification results. After deploying the model on mobile devices, YOLOv8n requires 7 GB of memory, with a processing capability of 8.0 GFLOPS, achieving a frame rate of 30 FPS. In contrast, TLI-YOLO, due to the iRMB attention mechanism, reduces memory usage to 6.3 GB while increasing the processing capability to 11.8 GFLOPS, maintaining a stable frame rate of 32 FPS. Additionally, a corresponding mini-program was developed, as shown in Figure 9b. Users upload images of rice diseases, which are then sent to cloud servers for identification using TLI-YOLO. Results are provided within one second under average network speeds, along with preventive and treatment measures for the disease.

## 4. Experiment and Results

### 4.1. Experiment Preparation

#### 4.1.1. Dataset

The dataset used in this study comprised two parts: the dataset for transfer learning and the rice disease dataset required for training the target model. The transfer learning dataset included the COCO128 and wheat disease datasets, while the dataset for the target model training was compiled through comprehensive searches on image search engines and photographs taken by us in specific locations. The wheat disease dataset is from the public dataset LWDCD2020 [56], which has about 12,000 images of nine classes of wheat diseases and one class of normal diseases that we collected.

The COCO128 dataset was selected for pretraining our model due to several key advantages. As a carefully curated subset of the larger Common Objects in Context (COCO) dataset, it maintains statistical representation of 80 diverse object categories while requiring significantly less computational resources. This diversity in objects, environments, and scales helps develop robust feature extractors that transfer effectively to rice disease detection tasks. The varied object scales in COCO128 particularly benefit the detection of disease symptoms ranging from small lesions to widespread patterns. Additionally, the bounding box annotation format aligned perfectly with our disease localization requirements, facilitating a seamless transition between the pretraining and fine-tuning phases. These empirical advantages, combined with COCO128’s status as a standard benchmarking dataset in the object detection community, justified its selection as the foundation for our transfer learning approach.

**Figure 10:**
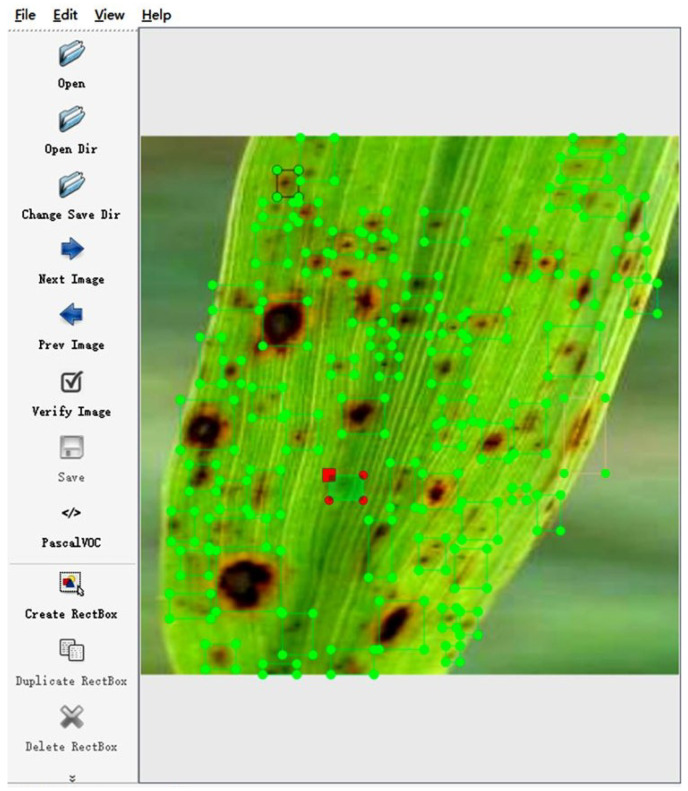
Annotation using LabelImg software.

To enhance the model’s robustness, we used three different devices for image capture and took photos from various distances (long, medium, and close range) to simulate more complex and realistic environments. The dataset was expanded to 1448 images through adjustments like white balance and brightness, and images were annotated using LabelImg software 1.8.6, as shown in Figure 10. We carefully analyzed the composition of the dataset, particularly the balance between different types of rice diseases. The labels were divided into three categories: bacterial blight, rice blast, and brown spot. These three categories were chosen because they are the most common and frequently occurring diseases in central China. The details of the dataset are shown in Table 1.

Regarding the issue of class imbalance, our dataset showed a relatively balanced distribution among the three disease categories. However, to address any potential imbalance, we applied oversampling for underrepresented categories and undersampling for overrepresented ones in the training set. These strategies were designed to mitigate the impact of class imbalance, which could otherwise cause the model to become biased and fail to generalize well across all disease types. Class imbalance can significantly affect model performance, leading to a bias toward majority categories, which may reduce accuracy for minority categories. By carefully managing class distribution, we aimed to enhance the model’s ability to accurately detect all types of rice diseases, thus improving overall model performance and reliability.

There may be potential biases in data collection, such as variations in image resolution and clarity due to different devices used for capturing the images. However, after resizing, all images were standardized to the same resolution and similar clarity. Additionally, environmental factors, such as differences in lighting and background complexity, could introduce biases that might cause the model to overfit. To mitigate these effects, we adjusted the brightness, applied data augmentation, and employed balancing techniques.

**Figure 11:**
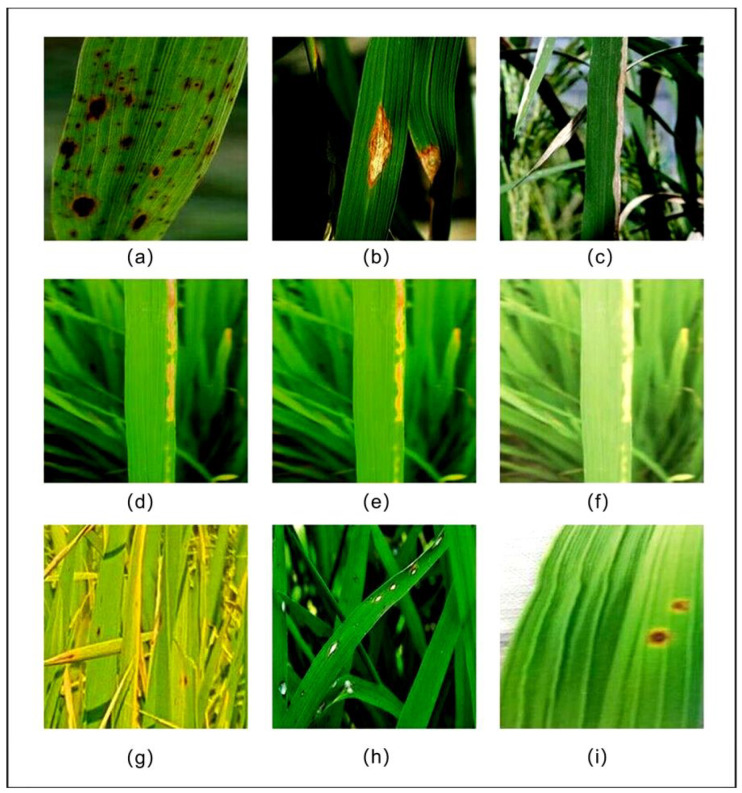
Representative images of the three diseases and their processing versions. (**a**–**c**) showcases representative images of brown spot, rice blast, and bacterial blight, respectively. (**d**–**f**) displays the original image, the result after brightness enhancement, and the outcome following white balance adjustment, respectively. (**g**–**i**) illustrates the results of capturing images of rice diseases from far, medium, and close distances, respectively.

Table 1 lists these categories. The dataset was split at an 8:1:1 ratio between the training, validation, and test sets, respectively. Figure 11 shows the classic legend of each disease and the processed version.

#### 4.1.2. Experimental Environment

Experiments were primarily conducted on Windows systems with GPU acceleration, while separate performance testing was carried out on Android devices to evaluate computational efficiency. Details on the computational resources required for model training and experimental parameters are elaborately presented in Table 2.

During the hyperparameter tuning process, we initially chose a smaller batch size due to the limited available GPU memory (only 6 GB). In our first attempt, we set the batch size to 16 but encountered an error: “Cuda out of memory”. We then reduced the batch size to eight, but when training our final model (as TLI-YOLO reached 12.6 GFLOPS), we again experienced memory overflow issues. As a result, we were forced to set the batch size to four.

The number of iterations was set to 200 because our model converged after approximately 200 epochs, making this the final choice for the epoch count. Given our constrained computational resources, we used grid search for hyperparameter selection. The learning rate was tested with values of [0.001, 0.01, 0.1], and the batch size options were [2, 4], while the epoch count was fixed at 200. The results are shown in Table 3. Based on the final outcomes, we determined that the model performed best when the batch size was four and the learning rate was 0.01.

#### 4.1.3. Evaluation Index

The target detection model’s performance was assessed in this research using the following metrics: GFLOPS, mAP, F1 score, precision, and recall. Precision measures the accuracy of an algorithm in detecting a single category [57], recall assesses whether all actual targets are detected, and the mAP calculates the average precision across different categories. The equations for the precision, recall, F1 score, AP, and mAP are provided in Equations (12)–(16):

(12) $$\mathrm{Precision}(\%)=\frac{TP}{TP+FP}\times 100\%$$

(13) $$\mathrm{Recall}(\%)=\frac{TP}{TP+FN}\times 100\%$$

(14) $$F1-\mathrm{score}(\%)=\frac{2\times \mathrm{Precision}\times \mathrm{Recall}}{\mathrm{Precision}+\mathrm{Recall}}$$

(15) $$AP=\int_{0}^{1}P\left(R\right)\;dR$$

(16) $$mAP=\frac{1}{\mathrm{SUM}}\sum_{k=1}^{\mathrm{SUM}}AP\left(k\right)$$

When the detection category is binary classification, the AP is equal to the mAP. In this paper, the mAP represents the mAP@.5.GFLOPS (giga floating point operations per second) measures a model’s computational capacity and training efficiency, indicating billions of floating point operations per second. It enables fair comparison of detection speeds across algorithms, independent of specific hardware or software.

### 4.2. Ablation Experiments

#### 4.2.1. Comparison of Ablation Experiments

The TLI-YOLO model developed in this study comprises the YOLOv8n baseline module, transfer learning steps, a small target detection layer module, WIOU loss function module, and the C2f_iRMB module that integrates an iRMB with C2f. This paper employed ablation experiments to analyze the contribution of each step or module. The experimental results are illustrated in Table 4. Comparing Model 1 and 2 with YOLOv8n demonstrates noticeable improvements in all aspects when transfer learning or a small object detection layer was added separately. Model 3, which combined the features of both Model 1 and Model 2, showed an increase in precision but a decrease in recall. The comparison between Model 4 and Model 3 reveals that incorporating the WIoUv3 loss function significantly enhanced all metrics, surpassing the performance of Model 1. Furthermore, the comparison between Model 5 and Model 3 indicates slight increases in the recall and mAP upon integrating the iRMB mechanism. Ultimately, our model, TLI-YOLO, exhibited a 7.8% increase in precision, a 7.2% increase in recall, a 7.6% increase in the mAP, and a 7.49% increase in F1 score compared with the YOLOv8n model. This success underscores the suitability of the TLI-YOLO model for rice disease detection tasks.

**Figure 12:**
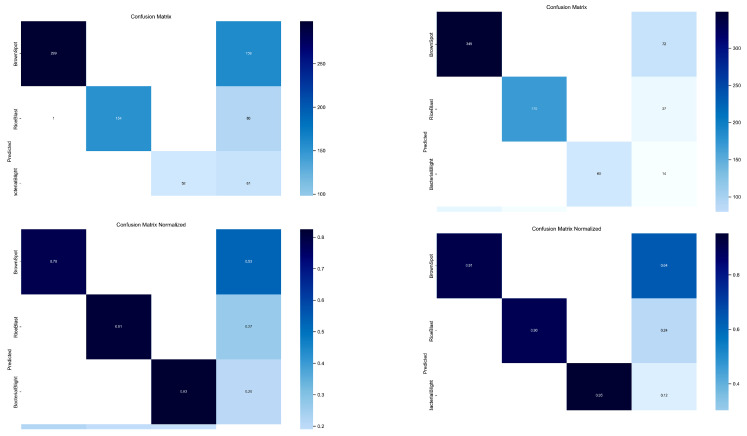
Diagram of confusion matrix.

In addition, common failure cases can be categorized into two types: false positive rate (misclassification) and missed detection rate. For YOLOv8n, the brown spot disease had the highest missed detection rate at 22% and a false positive rate of 0.58. In contrast, for the TLI-YOLO model, the missed detection rate for brown spot was reduced to 9%. The specific confusion matrix is shown in Figure 12.

After thorough analysis, we identified that the primary reason for this discrepancy is that brown spot typically represents small targets. YOLOv8n lacks sufficient focus on small target detection, leading to higher rates of both false positives and missed detections. However, by adding a dedicated small-target detection layer, TLI-YOLO shifted the model’s attention more toward small targets, significantly improving the detection performance for brown spot and reducing both the false positive and missed detection rates.

#### 4.2.2. Data Visualization

**Figure 13:**
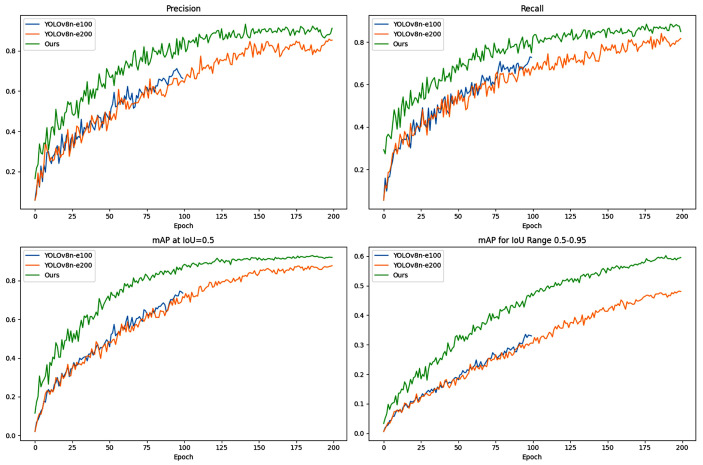
Comparison of evaluation metrics diagram.

This study conducted three sets of training examples to compare TLI-YOLO with YOLOv8n, including 100 and 200 iterations for YOLOv8n and 100 iterations for TLI-YOLO. As depicted in Figure 13, With just 100 iterations, TLI-YOLO was capable of achieving results comparable to those of YOLOv8n after 200 iterations.

**Figure 14:**
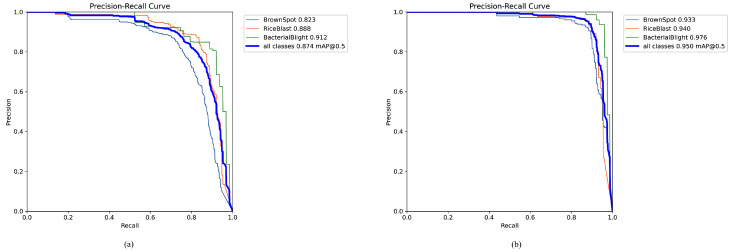
Comparison of PR curves diagram. (**a**) and (**b**) show the PR plots of YOLOv8n and TLI-YOLO, respectively.

This demonstrates the accelerated convergence speed of the improved model, significantly outperforming the original model within the same duration. Upon extending the improved model to 200 iterations, its metrics substantially exceeded those of the original model, indicating the superior effectiveness of the TLI-YOLO model. Furthermore, a comparison of the PR curves between TLI-YOLO and YOLOv8n is presented in Figure 14. Figure 14a represents YOLOv8n, and Figure 14b represents TLI-YOLO.

#### 4.2.3. Detection Visualization

**Figure 15:**
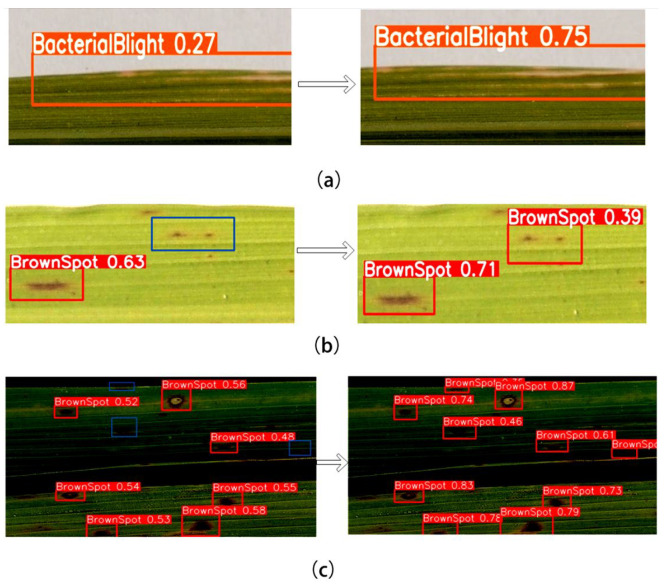
Visual comparison with other models, where blue squares indicate missed targets. For specific legend information, please refer to the article content.

In this study, both the TLI-YOLO and YOLOv8n models were used to infer the same rice disease images, with the inference results illustrated in Figure 14. The three images on the left show the results detected by the YOLOv8n model, while the three images on the right display the outcomes after detection by the TLI-YOLO model. The blue boxes in the images indicate missed targets. As indicated in Figure 15a, the TLI-YOLO model demonstrated significantly a higher detection accuracy compared with the initial model, especially when the detection targets had less-distinct features. Figure 15b,c reveals that when the detection targets were small and densely packed, the YOLOv8n model was prone to missed detections and low precision. In contrast, the TLI-YOLO model could easily detect small targets with a high degree of accuracy.

### 4.3. Comparison of Different Models

#### 4.3.1. Comparison of Consequences

To reliably assess the performance of the improved model, this study compared the proposed method with several current mainstream object detection methods. The results are shown in Table 5. The required training time and parameter number data compared with other models are shown in Table 5.

#### 4.3.2. Visualization of Comparison Results

**Figure 16:**
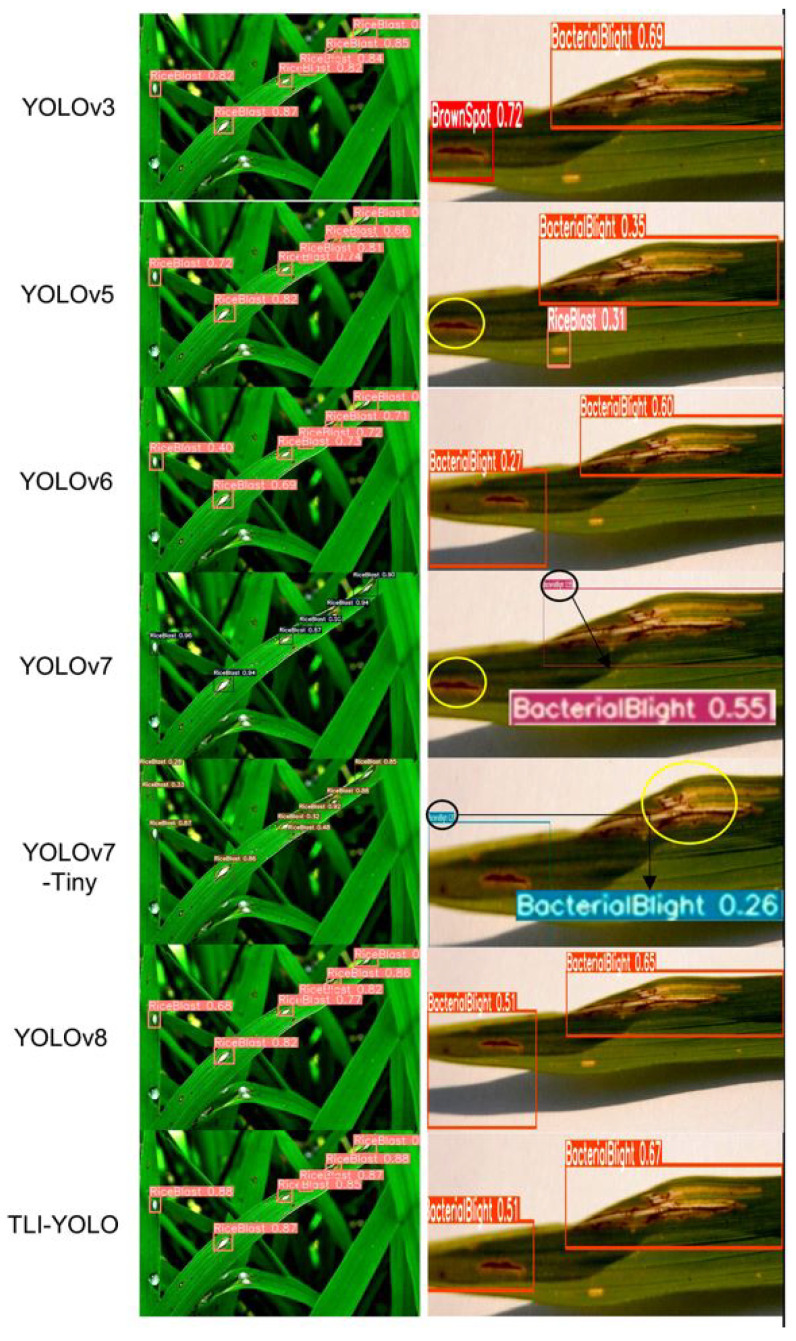
Visual comparison with other models, where yellow circles indicate missed targets.

This study conducted inference tests using the same image on the models with higher precision, as shown in Table 5 and illustrated in Figure 16. It was observed that the TLI-YOLO model’s confidence level for the same image was only slightly lower than that of YOLOv7 but significantly higher than the other methods. However, YOLOv7 has a critical flaw: It is prone to missing detections. Similarly, YOLOv5 and YOLOv7-Tiny also have issues with missed detections. YOLOv6 and YOLOv8 both exhibited lower confidence levels, whereas YOLOv3, despite not being the focus of this study, showed the best performance, but its GFLOPS count was nearly 23 times that of the improved model.

#### 4.3.3. Verification of Mobile Platform Detection

**Figure 17:**
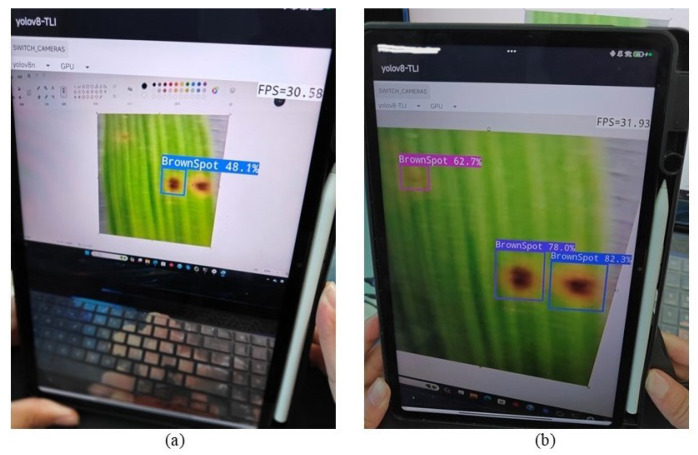
(**a**) The detection results using the YOLOv8n model. (**b**) The detection outcomes using the TLI-YOLO model.

**Figure 18:**
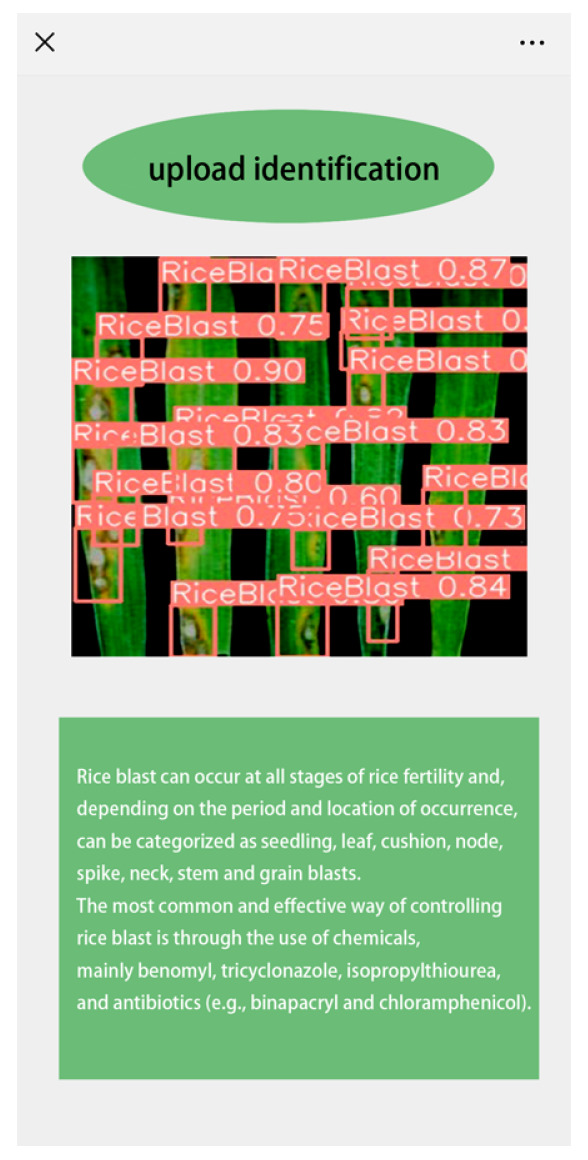
Mini-program.

In the final phase of the study, both the initial YOLOv8 model and the improved TLI-YOLO were deployed on the same Android device. Upon conducting object detection on the same image, it was observed that the modified model significantly outperformed the original in terms of both accuracy and detection efficiency. For a comparison of the results, refer to Figure 17. Subsequently, employing the second approach, the photo was uploaded to a server set up via Pycharm. The recognition process then took place on a PC, with the outcomes subsequently relayed back to the mobile application, where the disease categories, along with their descriptions and remediation strategies, were displayed. The detection results are illustrated in Figure 18. From the above content, it is clear that after deploying the model to the Android platform, the improved model significantly outperformed the initial model in terms of precision and had a low miss rate. Furthermore, when we conducted object detection tasks using a server, the mini-program could also clearly display the results. In conclusion, we can confirm that our model, once deployed on the Android platform, operates efficiently.

#### 4.3.4. Pretraining Visualization

**Figure 19:**
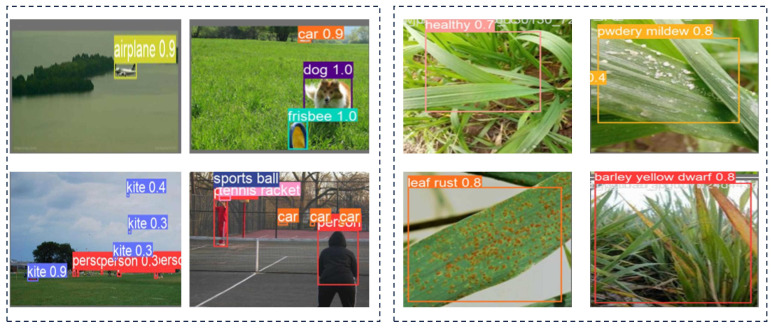
Pretraining visualized predictions.

To demonstrate the effectiveness of the transfer learning process, we provide a visual analysis of the predicted bounding boxes generated by the pretrained model. Figure 19 illustrates how the model, pretrained on the COCO128 dataset [49], accurately identified key regions of interest during the early prediction stage. These initial predictions are crucial for distinguishing between healthy and diseased plants in the fine-tuning phase. Additionally, Figure 19 shows the refined bounding boxes obtained after using the wheat disease dataset for auxiliary training, emphasizing the model’s improved precision in detecting plant diseases.

## 5. Conclusions

This paper presented TLI-YOLO, a target detection model tailored to rice disease recognition, aiming to address long training times and the need for large datasets while enabling real-time deployment on mobile devices.

To reduce training costs, the model leverages transfer learning, allowing it to learn from related tasks before fine-tuning on rice disease data. This reduces data demand and accelerates convergence, achieving an accuracy at 100 training rounds comparable to that of the baseline model at 200 rounds. This approach improved the accuracy by 3.7%, recall by 8.2%, and mAP by 6%.

To enhance detection of small disease targets, a small-target detection layer was added to the YOLOv8 architecture, improving the model’s focus on fine-grained features. This adjustment led to a 4.9% increase in precision, 3% increase in recall, and 2.8% increase in mAP.

For mobile deployment, particularly on Android devices, the iRMB module—suited for dense predictions—was integrated with the C2f module, forming the C2f_iRMB block. This improved computational efficiency while maintaining performance, bringing an additional 3.6% gain in precision, 3.7% in recall, and 3.3% in mAP.

The loss function was also updated from CIoU to WIoUv3, which introduces a dynamic focusing mechanism to reduce penalties for well-aligned boxes and suppress harmful gradients from poor anchors, thus enhancing the model’s generalization and robustness.

Future improvements will include knowledge distillation, dynamic pruning, and cross-crop detection. A teacher-student framework will allow a lightweight model to learn from TLI-YOLO, and contrastive learning will support adaptation across crops like rice, wheat, and corn.

Ultimately, TLI-YOLO showed a 7.8% increase in precision, 7.2% increase in recall, and 7.6% increase in mAP over YOLOv8n, with strong performance on the Android platform. The model was exported in ONNX and NCNN formats, enabling easy integration into agricultural systems, even without a Python environment.

Recent studies have shown that deep learning-based object detection models have made significant progress in rice disease recognition. For example, a UAV T-YOLO-Rice network based on Tiny YOLOv4 was proposed to improve the recognition ability of rice leaf diseases by integrating multiple modules (such as SPP, CBAM, and SCFEM), and 86% of the mAP was obtained on a dedicated dataset [58]. Another study constructed a YOLOv8_Rice model based on YOLOv8n, introduced an attention mechanism, deformable convolution, and a WISE IOU loss function, and achieved excellent performance in terms of accuracy and computational efficiency [59].

Compared with the above methods, the method proposed in this novel study proposes using transfer learning to quickly improve the detection speed and focuses on mobile terminal deployment to further save computing resources while maintaining accuracy.

Despite its strengths, the TLI-YOLO model exhibits some limitations, such as relatively large parameters, a slower inference speed, and a limited range of rice disease types it can detect. Our future research will focus on further streamlining the model and enhancing its inference speed. We also aim to refine the detection system by incorporating UAV imagery and diversifying the display of detection results on Android devices. To achieve these goals, we plan to reduce the weight of TLI-YOLO’s backbone network layers and optimize the SPPF module, which we anticipate will significantly decrease the model’s size and boost its detection capabilities. Furthermore, we intend to collect a broader range of rice disease datasets on-site and apply data augmentation techniques to expand the variety of rice diseases the model can recognize. Finally, by using pruning and distillation techniques, the model is further reduced in size and parameter count, enhancing the FPS and accuracy on mobile devices.

## Figures and Tables

**Table 1:** Detailed information on the dataset.

Datasets	Number (Images)	Category	Number (Category)
		Bacterial Blight	427
Train	1158	Rice Blast	352
		Brown Spot	379
		Bacterial Blight	61
Val	145	Rice Blast	43
		Brown Spot	41
		Bacterial Blight	56
Test	145	Rice Blast	41
		Brown Spot	48

**Table 2:** Hardware and software specifications.

Category	Name	Parameter
**Hardware**	PC	CPU: AMD Ryzen 5 7535H (Amd, CA, USA)
		Memory: 16 GB
		GPU: GeForce RTX 4050 Laptop (Nvidia, CA, USA)
		Graphics card: 6 GB
		OS: Windows 11 (Microsoft, Redmond, WA, USA)
	Android	CPU: Qualcomm Snapdragon Gen1 (Qualcomm Inc., San Diego, CA, USA)
		Memory: 8 G
		GPU: Adreno 730
		Graphics card: 1.05 G
		OS: Android 14
**Software**	Deep learning framework	PyTorch 2.2.1
	Programming languages	Python 3.9.18
	CUDA	11.8
**Hyperparameter**	Epochs	200
	Batch size	4
	Learning rate	0.01

**Table 3:** Results for the grid search method.

Learning Rate	Batch Size	Precision (%)	Recall (%)	mAP (%)
0.1	2	92.4	86.8	94.0
0.1	4	92.7	87.1	94.1
0.001	2	92.1	86.5	93.7
0.001	4	92.3	86.1	93.6
0.01	2	92.1	86.3	94.7
0.01	4	93.1	88.0	95.0

**Table 4:** Ablation experiment results.

Model	Transfer Learning	Detection Layer	WIoU	iRMB	Precision	Recall	mAP	F1
					(%)	(%)	(%)	(%)
YOLOv8n	×	×	×	×	85.3	80.8	87.4	83.0
Model 1	✓	×	×	×	89.0	**89.0**	93.4	89.0
Model 2	×	✓	×	×	86.5	83.4	89.6	84.9
Model 3	✓	✓	×	×	90.2	83.8	90.2	86.9
Model 4	✓	✓	✓	×	89.6	**89.1**	94.9	89.4
Model 5	✓	✓	×	✓	88.9	84.5	90.7	86.6
Ours	✓	✓	✓	✓	**93.1**	88.0	**95.0**	**90.5**

**Table 5:** Model comparison experiment results.

Model	Epochs	Precision (%)	Recall (%)	mAP (%)	F1 Score (%)	GFLOPS	Parameters	Time (Mins)
Faster RCNN	100	48.32	78.44	79.85	59.80	254.2	41,305,642	201
	200	50.39	84.23	81.78	63.06	283	103,694,777	406
YOLOv3	100	71.80	68.60	75.40	70.16	283	103,694,777	245
	200	88.32	84.90	91.60	86.58	283	103,694,777	460
YOLOv5	100	67.60	61.40	68.60	64.35	7.2	2,509,049	52
	200	79.60	74.20	82.40	76.81	11.9	4,238,441	103
YOLOv6	100	59.40	59.80	58.40	59.60	11.9	4,238,441	42.6
	200	74.60	68.50	78.00	71.42	13.2	6,020,400	86.7
YOLOv7	100	76.20	71.10	77.60	74.56	105.1	37,207,344	141
	200	87.30	84.50	90.30	85.88	105.1	37,207,344	283.3
YOLOv7-Tiny	100	75.10	80.80	81.80	77.85	13.2	6,020,400	109
	200	76.70	81.00	82.90	78.79	13.2	6,020,400	216.7
YOLOv10	200	88.60	85.2	91.00	86.87	6.7	2,300000	79
Ours	200	**93.10**	**88.00**	**95.00**	**90.48**	12.6	2,984,700	107

## Data Availability

Our data is not suitable for public release due to privacy.
